# Supplementary material for: Point Prevalence Survey of Antibiotic Use across 13 Hospitals in Uganda
Source: Antibiotics (Basel). 2022 Feb 4;11(2):199. doi: 10.3390/antibiotics11020199 (PMC8868487; doi:10.3390/antibiotics11020199)
Supplement: Supplementary file 1 [file antibiotics-11-00199-s001.zip › antibiotics-1543087-supplementary.pdf]

Table S1. Hospital characteristics

Hospital	Total beds	Ownership	Annual admissions	Included patients
Gulu RRH	347	Public	17,888	133
Hoima RRH	317	Public	17,455	103
Kagando	231	Private not-for-profit	12,168	61
Kiwoko	204	Private not-for-profit	9,810	43
Kumi	330	Private not-for-profit	4,642	47
Lacor	524	Private not-for-profit	16,839	168
Lira RRH	401	Public	13,421	119
Masaka RRH	333	Public	24,547	127
Moroto RRH	181	Public	7,864	99
Ruharo Mission	78	Private not-for-profit	2,870	6
Soroti RRH	251	Public	13,115	125
St. Anthony	93	Private not-for-profit	1,500	12
St. Francis Naggalama	100	Private not-for-profit	5,053	34

Table S2. Compliance with Uganda Clinical Treatment Guidelines by antibiotic

Antibiotic	Guideline compliance (*n*, %)
Amoxicillin	8 (30.8%)
Amoxyclav	47 (52.8%)
Ampicillin	2 (2.5%)
Ampicillin-cloxacillin	7 (36.8%)
Azithromycin	1 (20%)
Cef-sulbactam	0 (0%)
Cefazolin	1 (25%)
Cefixime	0 (0%)
Cefotaxime	0 (0%)
Ceftazidime	167 (32.6%)
Ceftriaxone	13 (28.9%)
Ciprofloxacin	0 (0%)
Clindamycin	13 (48.1%)
Cloxacillin	0 (0%)
Co-trimoxazole	1 (33.3%)
Doxycycline	1 (14.3%)
Erythromycin	53 (44.5%)
Flucamox	3 (20%)
Gentamicin	1 (25%)
Levofloxacin	86 (22.6%)
Meropenem	4 (40%)

Metronidazole	10 (62.5%)
Nitrofurantoin	0 (0%)
Penicillin	0 (0%)
Piperacillin-tazobactam	0 (0%)
Secnidazole	1 (100%)
Sulbactam	3 (100%)
Tinidazole	1 (100%)

Table S3. Adherence by hospital and antibiotic

**Percentage of prescribed antibiotic doses not administered to patients (*N*, %)**	
**Hospital**	
Gulu RRH	31 (21.4%)
Hoima RRH	0 (0%)
Kagando	0 (0%)
Kiwoko	0 (0%)
Kumi	0 (0%)
Lacor	2 (1.3%)
Lira RRH	52 (30.6%)
Masaka RRH	15 (7.2%)
Moroto RRH	10 (8.8%)
Ruharo Mission	0 (0%)
Soroti RRH	14 (8.9%)
St. Anthony	2 (10.5%)
St. Francis Naggalama	0 (0%)
**Antibiotic**	
Ampicillin	6 (6.7%)
Ampicillin-cloxacillin	24 (30.4%)
Ceftriaxone	25 (4.9%)
Ciprofloxacin	4 (8.9%)
Cloxacillin	1 (3.7%)
Co-trimoxazole	1 (33.3%)
Gentamicin	7 (5.9%)
Metronidazole	57 (15%)
Nitrofurantoin	0 (0%)
Penicillin	1 (6.3%)
Other[a]	0 (0%)
[a] Other includes: Amoxicillin, amoxyclav, azithromycin, cef-sulbactam cefazolin, cefixime, cefotaxime, ceftazidime, clindamycin, doxycycline, erythromycin, flucamox,	

levofloxacin, meropenem, nitrofurantoin, piperacillin-tazobactam, secnidazole, sulbactam, and tinidazole<br>Abbreviations: RRH = regional referral hospital

Table S4. Associations of antibiotic use with characteristics of females in the study sample

**Variable**	**Antibiotic use (*n* [%])**	**Odds ratio**	***p*-value**[1]
Age category			
<2 yrs	42 (82.9%)	1 (reference)	
2-50 yrs	332 (75.8%)	0.58	0.19
>50 yrs	47 (71.7%)	0.64	0.19
Hospital ownership			
Private not-for-profit	120 (62.8%)	1 (reference)	
Public	305 (73.0%)	1.60	<0.01*
Hospital			
Gulu RRH	51 (59.3%)	1 (reference)	
Hoima RRH	58 (86.6%)	4.42	0.09
Kagando	31 (83.8%)	3.55	<0.001*
Kiwoko	8 (36.4%)	0.39	0.01*
Kumi	16 (88.9%)	5.49	0.03*
Lacor	36 (44.4%)	0.55	0.06
Lira RRH	43 (66.2%)	1.34	0.39
Masaka RRH	68 (87.2%)	4.67	<0.001*
Moroto RRH	36 (72.0%)	1.76	0.14
Ruharo Mission	4 (80.0%)	2.75	0.38
Soroti RRH	49 (68.1%)	1.46	0.26
St. Anthony	8 (88.9%)	5.49	0.12
St. Francis Naggalama	17 (89.5%)	5.83	0.02*
Ward			
Maternal	210 (70.4%)	1 (reference)	
Medical	67 (71.1%)	0.80	0.35
Paediatric	75 (77.4%)	1.46	0.17
Surgical	73 (76.4%)	0.87	0.56
Underlying conditions			
HIV (no)	372 (67.5%)	1 (reference)	
HIV (yes)	19 (90.5%)	4.57	0.04*
TB (no)	384 (68.0%)	1 (reference)	
TB (yes)	6 (85.7%)	2.83	0.33
Malaria (no)	355 (68.1%)	1 (reference)	
Malaria (yes)	44 (73.3%)	1.29	0.41
COPD (no)	403 (69.6%)	1 (reference)	

COPD (yes)	3 (60.0%)	0.66	0.65
Malnutrition (no)	397 (68.8%)	1 (reference)	
Malnutrition (yes)	23 (92.0%)	5.21	0.03*
Hosp in past 90 days (no)	363 (67.8%)	1 (reference)	
Hosp in past 90 days (yes)	40 (50.0%)	1.90	0.08

[1] Statistical significance is noted by an "*" for all relationships with p <0.05.
Abbreviations: RRH = regional referral hospital; COPD = chronic obstructive pulmonary disease; HIV = Human immunodeficiency virus; TB = tuberculosis

Table S5. Associations of antibiotic use with characteristics of males in the study sample

**Variable**	**Antibiotic use (*n* [%])**	**Odds ratio**	***p*-value**[1]
Age category			
<2 yrs	60 (87.0%)	1 (reference)	
2-50 yrs	237 (75.7%)	0.47	0.04*
>50 yrs	70 (83.3%)	0.75	0.53
Hospital ownership			
Private not-for-profit	125 (69.4%)	1 (reference)	
Public	244 (84.7%)	2.44	<0.001*
Hospital			
Gulu RRH	33 (70.2%)	1 (reference)	
Hoima RRH	30 (83.3%)	2.12	0.17
Kagando	18 (75.0%)	1.27	0.67
Kiwoko	16 (76.2%)	1.36	0.61
Kumi	22 (75.9%)	1.71	0.59
Lacor	50 (57.5%)	0.57	0.04*
Lira RRH	43 (79.6%)	1.66	0.12
Masaka RRH	46 (93.9%)	6.51	<0.001*
Moroto RRH	45 (91.8%)	4.77	0.01*
Ruharo Mission	1 (100%)	NA	NA
Soroti RRH	47 (88.7%)	3.32	0.03*
St. Anthony	3 (100%)	NA	NA
St. Francis Naggalama	15 (100%)	NA	NA
Ward			
Maternal	9 (81.8%)	1 (reference)	
Medical	103 (75.7%)	1.03	0.65
Paediatric	113 (77.4%)	2.24	0.73
Surgical	144 (82.3%)	1.24	0.97
Underlying conditions			
HIV (no)	317 (77.3%)	1 (reference)	
HIV (yes)	24 (96.0%)	7.04	0.06

TB (no)	321 (77.3%)	1 (reference)	
TB (yes)	12 (92.3%)	3.51	0.23
Malaria (no)	312 (80.0%)	1 (reference)	
Malaria (yes)	41 (70.7%)	0.60	0.11
COPD (no)	349 (79.7%)	1 (reference)	
COPD (yes)	6 (85.7%)	1.53	0.70
Malnutrition (no)	341 (78.4%)	1 (reference)	
Malnutrition (yes)	26 (96.2%)	7.17	0.05
Hosp in past 90 days (no)	333 (79.3%)	1 (reference)	
Hosp in past 90 days (yes)	25 (80.6%)	1.09	0.86

[1] Statistical significance is noted by an "*" for all relationships with p <0.05.

Abbreviations: RRH = regional referral hospital; COPD = chronic obstructive pulmonary disease; HIV = Human immunodeficiency virus; TB = tuberculosis
